# Compensation or Restoration: Closed-Loop Feedback of Movement Quality for Assisted Reach-to-Grasp Exercises with a Multi-Joint Arm Exoskeleton

**DOI:** 10.3389/fnins.2016.00280

**Published:** 2016-06-21

**Authors:** Florian Grimm, Georgios Naros, Alireza Gharabaghi

**Affiliations:** Division of Functional and Restorative Neurosurgery, and Centre for Integrative Neuroscience, Eberhard Karls UniversityTuebingen, Germany

**Keywords:** robot-assisted rehabilitation, stroke rehabilitation, hemiparesis, motor recovery, upper-limb outcome assessment

## Abstract

Assistive technology allows for intensive practice and kinematic measurements during rehabilitation exercises. More recent approaches attach a gravity-compensating multi-joint exoskeleton to the upper extremity to facilitate task-oriented training in three-dimensional space with virtual reality feedback. The movement quality, however, is mostly captured through end-point measures that lack information on proximal inter-joint coordination. This limits the differentiation between compensation strategies and genuine restoration both during the exercise and in the course of rehabilitation. We extended in this proof-of-concept study a commercially available seven degree-of-freedom arm exoskeleton by using the real-time sensor data to display a three-dimensional multi-joint visualization of the user's arm. Ten healthy subjects and three severely affected chronic stroke patients performed reach-to-grasp exercises resembling activities of daily living assisted by the attached exoskeleton and received closed-loop online feedback of the three-dimensional movement in virtual reality. Patients in this pilot study differed significantly with regard to motor performance (accuracy, temporal efficiency, range of motion) and movement quality (proximal inter-joint coordination) from the healthy control group. In the course of 20 training and feedback sessions over 4 weeks, these pathological measures improved significantly toward the reference parameters of healthy participants. It was moreover feasible to capture the evolution of movement pattern kinematics of the shoulder and elbow and to quantify the individual degree of natural movement restoration for each patient. The virtual reality visualization and closed-loop feedback of joint-specific movement kinematics makes it possible to detect compensation strategies and may provide a tool to achieve the rehabilitation goals in accordance with the individual capacity for genuine functional restoration; a proposal that warrants further investigation in controlled studies with a larger cohort of stroke patients.

## Introduction

Assistive rehabilitation technology allows an increase and standardization in the amount of upper limb movement therapy after stroke, potentially resulting in improved arm/hand function and muscle strength, albeit respective trials have, as yet, provided only low-quality evidence (Kwakkel et al., 2008; Mehrholz et al., 2015). In clinical settings, therapists often provide the patient with feedback on the movement quality to encourage relearning of premorbid movement patterns (Cirstea and Levin, 2007). Particularly in patients with severe impairment, deficits in the range and coordination of elbow and shoulder movements might interfere with reaching performance (Cirstea and Levin, 2000; Cirstea et al., 2003). Moreover, motor compensation could limit gains in motor function by learned non-use and lead to pain and joint contractures in the long run (Cirstea and Levin, 2007). However, although robot-assisted therapy focuses on task performance, it usually does not differentiate between compensation strategies and genuine motor restoration despite being capable of objective movement evaluation (Kwakkel et al., 2008). Although kinematic parameters would be particularly suitable for assessing movement quality during rehabilitation exercises, current robotic devices tend to capture end-point measures that lack information on proximal interjoint coordination (Nordin et al., 2014) which would be necessary to differentiate recovery from compensation. In this context, a gravity-compensating multi-joint exoskeleton could not only support reach-to grasp movements in severely affected stroke patients but also provide closed-loop virtual reality feedback of movement quality during task-oriented training. This pilot study intended to explore the methodological feasibility and clinical validity of virtual reality visualization and closed-loop feedback of joint-specific movement kinematics to capture the evolution of upper extremity movement patterns in severely affected stroke patients. We furthermore wanted to quantify the individual degree of natural movement restoration or compensation for each patient. When a proof-of-concept is demonstrated here, such an approach would provide a tool to follow rehabilitation goals in accordance with the individual capacity for genuine functional restoration, a strategy that could then be verified by further investigations in controlled studies.

## Materials and methods

We recruited ten right-handed healthy subjects (6 males, mean age: 29 ± 4 [24 39] years) and three right-handed stroke patients (all male, mean age: 62 ± 6 [56 68] years). The patients were in the chronic phase after stroke (57 ± 22 [34 78] months) and presented with a severe and persistent hemiparesis of the left side. To ensure that our results were comparable to earlier studies, *coordination, speed* and *reflexes* were not taken into account. This resulted in a modified upper extremity Fugl-Meyer-Assessment scores (UE-FMA) of 12, 12, and 25, respectively. This study was in accordance with the guidelines of the ethic committee of the local medical faculty. Participants performed either a single session (healthy control group) or 20 sessions in the course of 4 weeks (patients) of reach-to-grasp training with a multi-joint exoskeleton attached to the left arm. The orthosis was calibrated according to the individual anatomy (e.g., shoulder position, forearm/upper arm length) of each patient. This setup and calibration of the system before every session took about 5 min per patient. Each session lasted approximately 30 min and consisted of 150 trials. The general experimental setup has already been described in detail elsewhere (Grimm and Gharabaghi, 2016; Grimm et al., under review) and is cited here when applied in the same way.

### Exoskeleton and virtual reality

**Figure 1:**
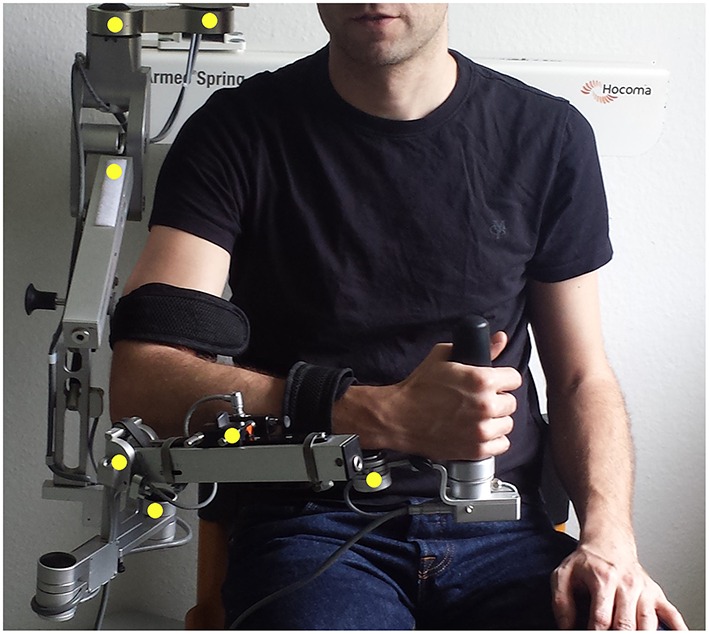
**Exoskelleton setup and location of angle sensors within the device (yellow dots)**.

We used a commercially available (Armeo Spring, Hocoma, Volketswil, Switzerland) rehabilitation exoskeleton for shoulder, elbow and wrist joints with seven axes (i.e., degrees of freedom) providing antigravity support for the paretic arm and registration of movement kinematics and grip force. This device allowed individual adjustments e.g., of gravity compensation, thereby supporting patients with severe impairment in performing task-oriented practice within a motivating virtual environment. Kinematic sensor data was provided by 7 built-in angle sensors (sensor resolution <0.2°) for shoulder flexion/abduction (1 sensor), shoulder rotation (2 sensors), elbow flexion/extension (1 sensor for horizontal registration, 1 sensor for vertical registration), forearm pronation/supination (1 sensor) and wrist flexion/extension (1 sensor). The shoulder rotation was calculated as the sum of the two sensors. The upper arm movement was calculated as the angle between forearm and upper-arm in three-dimensional space. The sensors were placed directly in the movement axis of the exoskeleton within the joints, allowing an accurate registration of the actual joint position of the upper arm, forearm and hand (Figure 1). Kinematic data of hand closure/opening could not be captured directly with this set up. We therefore estimated the hand function indirectly by registering the grip force. Grip force has previously been shown to correlate with motor function in chronic stroke patients (Boissy et al., 1999) and was captured with an in build mid-palmar grip pressure sensor in the present study.

**Figure 2:**
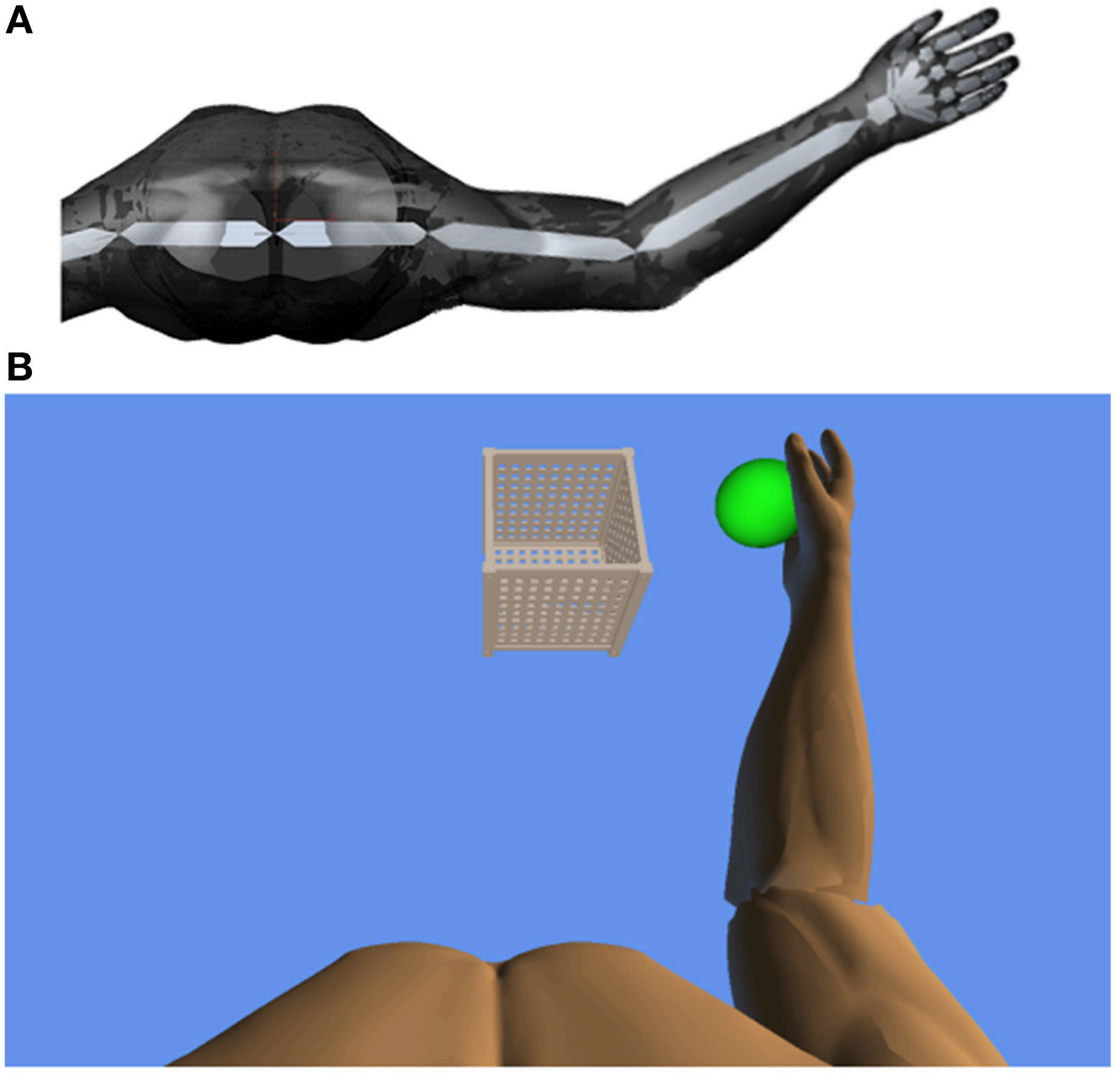
**(A)** Bone architecture of the three-dimensional multi-joint visualization of the user's arm in virtual reality. **(B)** Virtual training environment for reach-to-grasp movements toward a ball which changes its position in space after each trial. The ball has to be grasped, carried to a distant basket and then released again.

Thereby, a complete real-time registration of the subject's kinematic reach-to-grasp movement could be performed with the orthosis. We extended these features in-house by using the real-time sensor data of the exoskeleton to display a three-dimensional multi-joint visualization of the user's arm in virtual reality (Figure 2A). The exercises where displayed to the subjects on a monitor in front of the setup. For this purpose, we captured the angles of all arm joints and the grip force from a shared memory block using a file mapping communication protocol. The virtual arm engine was programmed in a Microsoft XNA™ framework. The arm model utilized by the engine was constructed as a meshed bone-skin combination with 54 bones (3Ds Max 2010™, Autodesk). The measured joint angles and grip forces of the device were used to modify the bone-vectors of the meshed model according to the movements of the user thereby providing online closed-loop feedback. The kinematic data and the 3D virtual representation were updated in 20 ms intervals. The joint angles of the exoskeleton were directly represented in virtual reality, whereas the grip forces were augmented to feedback natural hand function. Prior to each session, participants were instructed to perform a natural reach-to-grasp movement during the task by using distal (elbow) rather than proximal (shoulder) movements. The participants were moreover encouraged to track and adjust their movements accordingly with the information provided by the virtual environment. Furthermore, they were informed that their movement quality would be captured and evaluated afterwards. This preparation was intended to prime the participants to exploit the information provided by the virtual feedback. The three-dimensional visualization of the arm was then applied during each task as an implicit online feedback of movement quality, since explicit information can disrupt motor learning in stroke patients (Boyd and Winstein, 2004; Cirstea and Levin, 2007). Various virtual training paradigms were designed to allow for different rehabilitation exercises resembling activities of daily living.

### Task design

In this study, participants performed a reach-to-grasp movement toward a ball which changed its position in virtual space after each trial, necessitating three-dimensional transfer movements. The ball had to be grasped, carried to a distant basket and then released again (Figure 2B). The virtual hand could interact with the ball as soon as it entered a defined range around the latter. The ball changed its color according to the hand position (white: out of range, green: possible to grasp, yellow: possible to transfer, red: possible to release). The grasping and releasing of the virtual ball was performed by applying force to the grip sensor and opening the hand, respectively, while the threshold was adjusted to the individual strength of the user. No other support was provided during the exercises. The level of orthotic assistance remained constant in the course of the 20 sessions.

### Outcome measures

The kinematic assessment included both motor performance and movement quality (Nordin et al., 2014). The motor performance was estimated with regard to accuracy, temporal efficiency and range of motion. Movement accuracy, more specifically the decrease of inaccuracy, was captured by calculating changes of movement direction along an optimal path toward the targets, by estimating the distance function between the hand-position and the final endpoint, and by calculating the second derivative of the function to acquire the number of turning points for each task (Cirstea et al., 2006). Temporal efficiency was captured as the mean velocity of the hand between the targets while calculating their distance for X-, Y- and Z-directions in virtual units (vu). The range of motion of each joint was measured according to the orthosis and displayed in degrees along with the mean change in grip pressure. Movement quality of proximal inter-joint coordination was defined as the amount of compensatory shoulder inward rotation during the task and quantified by a shoulder/elbow index, i.e., the degree of inward rotation of the shoulder in relation to the degree of elbow movement. More specifically, a larger proportion of shoulder movement would indicate compensation, while a larger proportion of elbow movement for the same task would indicate a rather natural movement.

### Statistics

Statistical analysis was performed on a Matlab 2010b Engine. Data was tested for linear distribution using the Lilliefors-test (2-sided goodness-of-fit test). The non-parametric Kruskal–Wallis was used for group comparisons. To estimate the evolution of parameters during training, a robust multilinear regression model was fitted. Although the Lilliefors-test revealed normality of the data, a robust multilinear regression analysis was applied in order to minimize the impact of outliers. The fitting function was based on an iteratively reweighted least squares algorithm. The weights of each iteration were calculated by applying a bisquared function to the residuals of the previous iteration. For every fitting function the slope *b* of coefficient estimates was presented. The significance level was set to *p* = 0.05 for all tests.

## Results

Patients differed significantly with regard to motor performance (accuracy, temporal efficiency, range of motion) and movement quality (proximal inter-joint coordination) from the healthy control group (Table 1). Most notably, they applied compensatory strategies by using more shoulder than elbow movements.

**Table 1:** **Overview of kinematic data for subjects and patients, respectively**.

**Parameter**	**Subjects**	**Patients**	***p*-value**
Inaccuracy, number of turning points	4.20 ± 0.42 [4.00 5.00]	8.75 ± 2.51 [6.00 13.00]	<0.001
Average velocity (distance/time) (vu/s)	13.86 ± 2.17 [11.31 17.53]	3.89 ± 1.85 [0.90 8.04]	<0.001
Grip pressure	0.45 ± 0.19 [0.14 0.69]	0.684 ± 0.27371 [0.22 1.04]	<0.001
Shoulder movement, angle in degrees (°)	32.90 ± 8.03 [21.81 44.42]	22.00 ± 12.48 [11.93 35.97]	<0.001
Elbow movement, angle in degrees (°)	36.83 ± 7.65 [19.65 44.79]	19.18 ± 5.03 [6.94 28.87]	<0.001
Shoulder/elbow index	0.78 ± 0.05 [0.71 0.88]	1.36 ± 0.30 [1.0 1.58]	<0.001

However, the patients showed motor learning in the course of the training program with significant changes in most kinematic measures toward the reference parameters of healthy participants (Table 2) paralleled by improved FMA-UA scores (+1, +2, +5 points, respectively) in the end of the training.

**Table 2:** **Individual slopes of robust multilinear regression models of kinematic changes in the three stroke patients (n.s.: not significant)**.

**Parameter**	**Patient 1**	**Patient 2**	**Patient 3**
Inaccuracy, turning points	−0.24, *p* = 0.26 (n.s.)	−0.14, *p* = 0.02	−0,06, *p* < 0.001
Average velocity (distance/time) (vu/s)	+ 0.14e-3, *p* < 0.001	+ 0.13e-3, *p* < 0.001	+ 0.17e-3, *p* < 0.001
Grip pressure	+1.1e-3, *p* = 0.08 (n.s.)	+4.4e-3, *p* < 0.001	+8.4e-3, *p* < 0.001
Shoulder movement, angle in degrees (°)	+0.14, *p* = 0.45 (n.s.)	+0.4, *p* = 0.01	+1.2, *p* < 0.001
Elbow movement, angle in degrees (°)	+0.49, *p* = 0.01	+0.36, *p* = 0.01	+0.36, *p* < 0.001
Shoulder/elbow index	−27e-3, *p* = 0.007	−15e-3, *p* = 0.05	−6e-3, *p* = 0.46 (n.s.)

**Figure 3:**
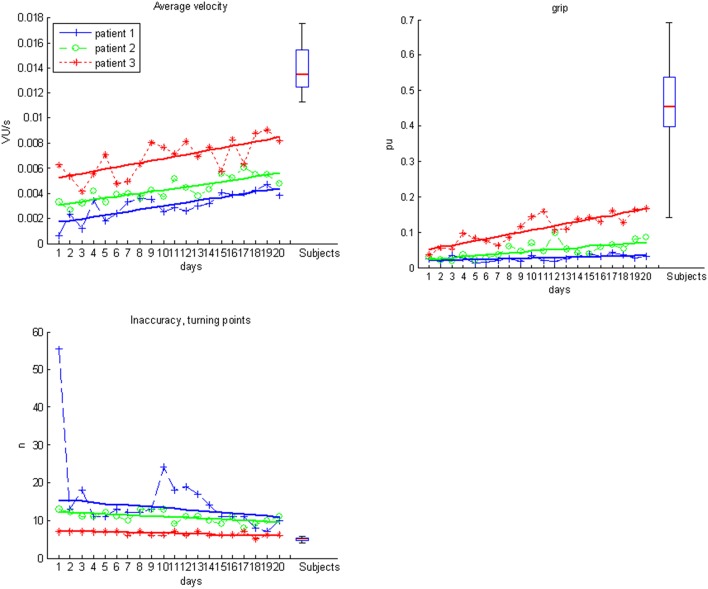
**Motor performance data for subjects (boxplots) and individual patients over the time course of training**.

**Figure 4:**
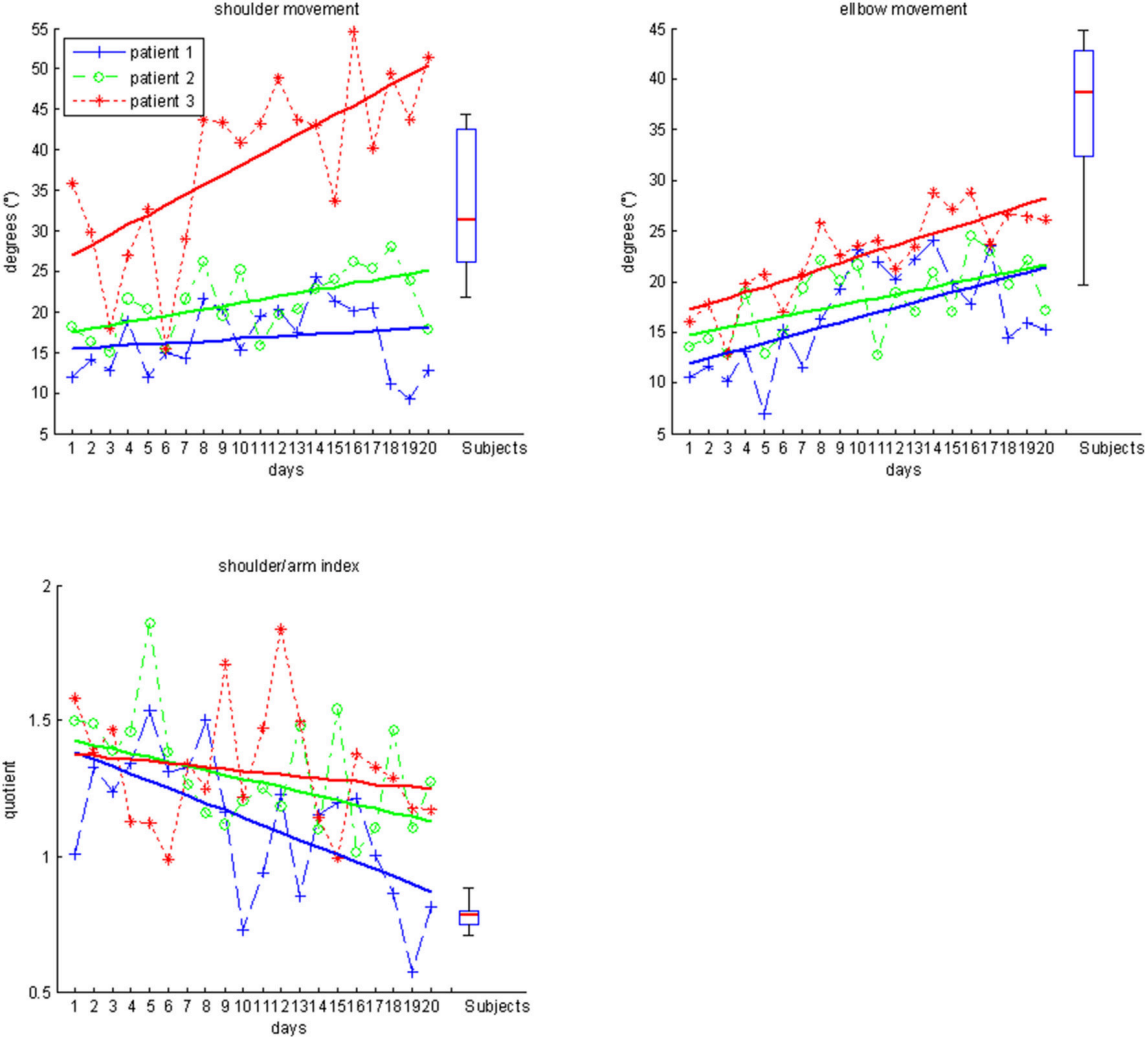
**Movement quality data for subjects (boxplots) and individual patients over the time course of training**.

Most importantly, the evolution of movement pattern kinematics of the shoulder and elbow enabled us to quantify the individual degree of natural movement restoration for each patient: Patient 1 had the lowest scores in all kinematic parameters and also showed the poorest motor performance (Figure 3). However, he presented with the steepest evolution of movement quality and was the only patient to reach the reference parameter of healthy participants (Figure 4). By contrast, patient 3 showed the highest kinematic parameters, i.e., the best motor performance (Figure 3), but also revealed the strongest compensatory movements with the shoulder (Figure 4).

## Discussion

Rehabilitation devices with a gravity-compensating arm exoskeleton provide assistance for intensive exercises in severely affected stroke patients and may thereby improve motor performance in the course of a training intervention (Housman et al., 2009). However, functional gains in hemiparetic patients are often achieved by non-physiologic movements with a disturbed shoulder-arm inter-joint coordination (Levin, 1996; Levin et al., 2002). Although these compensatory strategies might be efficient in short-term task accomplishment, they may lead to long-term complications such as pain and joint-contracture (Cirstea and Levin, 2007). Movement pattern kinematics may provide accurate, valid, reproducible and predictive measures of the impairment severity in chronic stroke (Subramanian et al., 2010) and of atypical movement patterns that aim to compensate the diminished range of motion of the affected limb (Cirstea and Levin, 2000). In this context, providing detailed information about how the movement is carried out, i.e., the movement quality regarding inter-joint coordination, is more liable to recover premorbid movement patters and to avoid compensatory movements than to provide information about movement outcome, i.e., end-point based accuracy information, only (Cirstea et al., 2006; Cirstea and Levin, 2007). When this feedback is administered during virtual reality training, even less compensation was achieved in the moderate-to-severe group (Subramanian et al., 2013). However, in these previous studies, information on movement quality was provided *explicitly* to the patients via auditory feedback. Moreover, all patients who received this feedback on their movement pattern kinematics were *mildly or moderately-to-severely* affected and were able to perform reach-to-grasp movements without assistance.

In the present feasibility study, we extended this line of research by incorporating information on movement quality as *implicit* closed-loop feedback in the virtual environment of an exoskeleton-based rehabilitation device suitable for *severely affected* stroke patients who require gravity-support to perform activities of daily living such as reach-to-grasp exercises. Notably, antigravity-support did not interfere with the kinematic evaluation of proximal inter-joint coordination. By contrast, this approach allowed disentangling in patients with severe impairments whether improved motor performance was achieved by compensation or by functional restoration. Notably, improvement in kinematic measures may be misleading since driven by compensatory strategies. The observations of this study highlighted that these measures were not sufficient to fully assess the evolution during motor rehabilitation thereby supporting the analysis of multi-joint information along the movement trajectory. Moreover, the continuous visual feedback of the whole arm kinematics allowed the patients to adjust their movement quality online during each task; an approach closely resembling natural motor learning. Although pathological measures improved significantly toward the reference parameters of healthy participants, this study did not provide evidence for the specificity of these effects to the implemented setup, i.e., feedback modality. Future studies need therefore to address this question by directly comparing multi-joint with end-effector feedback in controlled trials with long-term follow up evaluation, before conclusions about the therapeutic superiority of the presented approach can be drawn. In any case, however, the diagnostic advantage of detecting compensatory strategies (i.e., use of proximal instead of distal joints in a reach-to-grasp task) with the help of the multi-joint orthosis remains evident.

Future studies may explore the additional effects of brain stimulation on movement quality for assisted reach-to-grasp exercises: a recent study which applied bilateral transcranial direct current stimulation has demonstrated improved motor performance beyond the natural learning curve while using the very same multi-joint arm exoskeleton studied in the present work (Naros et al., 2016a). Moreover, brain state-dependent transcranial magnetic stimulation has been demonstrated to induce robust increases of corticospinal excitability (Kraus et al., 2016b) and may thereby amplify use-dependent plasticity when applied in conjunction with orthotic rehabilitation devices (Gharabaghi, 2015). Future approaches may also address patients with even more limited residual motor function as well (which might not benefit from the presented approach) by providing closed-loop feedback with a *robotic* multi-joint exoskeleton during brain-states in which both the participant's effort to move and the responsiveness of the brain for peripheral input are reflected (Brauchle et al., 2015). In such a restorative framework, closed-loop interfaces follow an operant conditioning rationale, providing contingent feedback to facilitate self-regulation of specific brain activity which is considered to be beneficial for recovery and might ultimately lead to functional gains (Bauer and Gharabaghi, 2015a). Accordingly, these brain-robot interfaces were found to constitute a back-door to the motor system (Bauer et al., 2015; Gharabaghi et al., 2014a), since this type of feedback training may result in connectivity changes of cortico-spinal (Kraus et al., 2016a) and cortico-cortical motor networks (Vukelić et al., 2014; Vukelić and Gharabaghi, 2015a,b) and thereby lead to behavioral gains after the intervention (Naros et al., 2016b). Recently, pilot data has suggested that such restorative brain-robot interfaces may even lead to task-specific motor improvement in chronic stroke (Naros and Gharabaghi, 2015).

Problematic for restorative approaches is, however, that the considerable challenge of these devices (Bauer and Gharabaghi, 2015b; Fels et al., 2015) might condition the patients to explore alternative, i.e., therapeutically non-desired, strategies (Gharabaghi et al., 2014b). Particularly in patients with severe impairments, motor compensation could limit genuine motor restoration. In this context, detection and closed-loop feedback of movement quality during rehabilitation exercises would allow differentiating recovery from compensation and thus encourage the relearning of premorbid movement patterns. For those patients, however, who benefit less from the implicit closed-loop information provided in the presented set-up (e.g., patient 3) more explicit feedback or even segmental movement restriction by the orthosis might be necessary to reinforce the targeted movement pattern.

In conclusion, virtual reality visualization and feedback of joint-specific movement kinematics facilitates to monitor the evolution of upper extremity movement kinematics and to quantify the individual degree of natural movement restoration in the course of rehabilitation training of severely motor impaired patients; controlled studies with a larger cohort of stroke patients need to investigate whether this approach also allows to achieve the rehabilitation goals in accordance with the individual capacity for functional recovery.

## Author contributions

FG participated in the study design and software development, supervised the measurement sessions and carried the data analysis. GN supervised the measurement sessions. AG participated in the study design and data analysis. Authors jointly drafted and approved the final manuscript.

### Conflict of interest statement

The authors declare that the research was conducted in the absence of any commercial or financial relationships that could be construed as a potential conflict of interest.
